# Thermolysis-Driven Growth of Vanadium Oxide Nanostructures Revealed by *In Situ* Transmission Electron Microscopy: Implications for Battery Applications

**DOI:** 10.1021/acsanm.3c00397

**Published:** 2023-05-03

**Authors:** Dnyaneshwar S. Gavhane, Atul D. Sontakke, Marijn A. van Huis

**Affiliations:** † Soft Condensed Matter and Biophysics, Debye Institute for Nanomaterials Science, Utrecht University, Princetonplein 5, Utrecht 3584 CC, The Netherlands; ‡ Condensed Matter and Interfaces, Debye Institute for Nanomaterials Science, Utrecht University, Princetonplein 5, Utrecht 3584 CC, The Netherlands

**Keywords:** *in situ* transmission electron microscopy, thermolysis, 2D materials, V_2_O_5_, VO_2_, *ex situ* growth

## Abstract

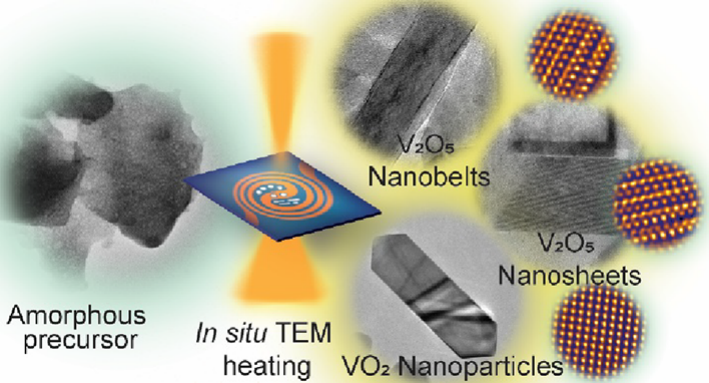

Understanding the growth modes of 2D transition-metal oxides through direct observation is of vital importance to tailor these materials to desired structures. Here, we demonstrate thermolysis-driven growth of 2D V_2_O_5_ nanostructures via *in situ* transmission electron microscopy (TEM). Various growth stages in the formation of 2D V_2_O_5_ nanostructures through thermal decomposition of a single solid-state NH_4_VO_3_ precursor are unveiled during the *in situ* TEM heating. Growth of orthorhombic V_2_O_5_ 2D nanosheets and 1D nanobelts is observed in real time. The associated temperature ranges in thermolysis-driven growth of V_2_O_5_ nanostructures are optimized through *in situ* and *ex situ* heating. Also, the phase transformation of V_2_O_5_ to VO_2_ was revealed in real time by *in situ* TEM heating. The *in situ* thermolysis results were reproduced using *ex situ* heating, which offers opportunities for upscaling the growth of vanadium oxide-based materials. Our findings offer effective, general, and simple pathways to produce versatile 2D V_2_O_5_ nanostructures for a range of battery applications.

## Introduction

Vanadium pentoxide (V_2_O_5_), the semiconductor in the transition-metal oxides family with a two-dimensional (2D)-layered structure, has attracted significant attention from the scientific community. Featuring high theoretical capacity, low cost, abundant source, good safety, and easy preparation methods, V_2_O_5_ has attracted enormous interest in rechargeable batteries.^1−5^ Because of the typical lamellar crystal structure of orthorhombic V_2_O_5_, it has been extensively studied for application as a lithium-ion battery cathode material.^6−8^ Besides this, V_2_O_5_ has also attracted great attention as a potential candidate for catalysts^9^ and gas sensor applications.^10−12^

Considering its exceptional and versatile role in various applications, a variety of synthesis methods to prepare V_2_O_5_ nanostructures has been developed including hydrothermal/solvothermal synthesis, sol–gel processing, template-based methods, electrochemical deposition, etc.^13^ Nanostructures of V_2_O_5_ consist of 1D nanorods,^14,15^ nanobelts,^16,17^ nanofibers,^18^ 2D nanosheets,^19−23^ 3D hollow porous,^24,25^ and hierarchical^26,27^ nanostructures. One-dimensional V_2_O_5_ nanostructures are prepared mostly with sol–gel and hydrothermal methods, which are relatively simple, using a V_2_O_5_ powder or V_2_O_5_ crystals as a precursor. Two-dimensional nanosheets are prepared with the liquid exfoliation of bulk V_2_O_5_ crystals^20^ and the hydrothermal method.^28^ All of these V_2_O_5_ nanostructures that are grown through different methods are continuously being used in rechargeable battery applications.

Thermolysis of ammonium metavanadate (AMV), an amorphous single solid-state precursor, produces crystalline V_2_O_5_ structures.^29−32^ Decomposing AMV precursor is a quite straightforward and feasible method to produce V_2_O_5_ nanostructures with no complex requirements or conditions. This unique and simple synthesis method has not been explored significantly compared to the conventional routes. An exploration of the underlying growth mechanisms of V_2_O_5_ nanostructures through the thermolysis of an amorphous single solid-state precursor provides an extraordinary opportunity to grow these nanostructures with great control. Real-time observations at the atomic scale of various physical and chemical processes of phase transitions,^33−36^ growth,^37−39^ and sublimation^40−42^ can be achieved by *in situ* transmission electron microscopy (TEM) with high spatial and temporal resolution. The *in situ* TEM technique has been extensively used to study the dynamic processes in various materials.^34,43−49^*In situ* TEM growth of MoS_2_ through thermolysis of a single solid-state precursor was observed in several experiments.^50−52^ In other experiments, 2D WS_2_ vertical and horizontal layers were grown in TEM through thermal decomposition of a single solid-state precursor.^53,54^ Metal oxide nanomaterials were also observed to grow during *in situ* TEM experiments.^55−57^

In this work, with the combination of *in situ* TEM heating and thermolysis of ammonium metavanadate (NH_4_VO_3_) inside the TEM, we have observed the real-time growth of crystalline V_2_O_5_ nanostructures on the SiN_*x*_ membrane of the heating chip. The heating of the precursor inside the TEM shows multiple growth stages to finally yield the shape of crystalline V_2_O_5_ nanostructures. The formation of mesoporous structures on an amorphous precursor due to the removal of NH_3_ and H_2_O to the crystallization at a small scale was observed in real time during the thermolysis. Two different types of final nanostructures are observed during this *in situ* TEM thermolysis process for the V_2_O_5_; 1D nanobelts and 2D nanosheets. The phase transformation of 2D V_2_O_5_ to 3D VO_2_ nanostructures is also observed at elevated temperatures in the *in situ* TEM experiment. All these intermediate processes cannot be observed and controlled by means of conventional growth methods, while with *in situ* TEM, it is possible to observe these in real time and to gain control over the different growth stages of V_2_O_5_ nanostructures to obtain the desired materials. These *in situ* TEM results can be used to fine-tune the growth parameters during the thermolysis to design V_2_O_5_ nanomaterials, which are highly relevant for rechargeable battery applications.

## Experimental Section

### *In Situ* Transmission Electron Microscopy

*In situ* TEM heating experiments were performed on a DENSsolutions heating holder and Wildfire S3 heating chip with electron transparent ∼30 nm thick SiN_*x*_ windows. Before the *in situ* TEM heating, the samples were prepared by dissolving high purity NH_4_VO_3_ (AMV) (Sigma-Aldrich, 99.9%) in ethanol to form 5 vol % solutions. These solutions were sonicated for 15 min, drop cast onto the plasma cleaned heating chip, and dried in air. The heating chip was then introduced into an FEI Talos F200X TEM operated at 200 kV for imaging. In all the TEM heating experiments, samples were preheated to 100 °C for at least 10 min to remove organic residues. The temperature was then increased from 100 to 700 °C in steps of 20 °C. During *in situ* TEM heating, after each 100 °C, the temperature was held constant to observe and image growth changes in the first pilot experiment. The TEM heating experiments were repeated multiple times. Scanning TEM (STEM) imaging on the FEI Talos F200X was conducted using a probe current of 30 pA and a dwell time per pixel of 4.0 μs. All EDS chemical mapping experiments were performed on the Talos F200X TEM equipped with a Chemi-STEM elemental analysis setup. Each of the EDS maps was recorded for 15 min to improve the signal-to-noise ratio. The stoichiometry of the vanadium oxides was determined by EDS quantification, using a standard software package supplied with a TalosF200X microscope (TFS software). The Cliff-Lorimer method was used for EDS quantification. The spatial resolution of the *in situ* set-up is 1.2 Å (the resolution of the TalosF200X microscope is not degraded at elevated temperature), while the temporal resolution both in TEM and STEM corresponds to a recording rate of 20 frames per second or higher. For all the filtered images, the contrast was improved between the material and amorphous region in the background by applying a mask on the amorphous region followed by the inverse fast Fourier transformation (IFFT) for better display purposes. The TEM imaging simulations were performed using QSTEM software. The simulated image was generated with the following settings: accelerating voltage: 200 kV, objective aperture: 15 mrad, convergence angle: 1 mrad, focal spread: 2 nm, defocus: 10.

### *Ex Situ* Experiments and Characterization

The *ex situ* heating experiments were performed in a vacuum oven Nabertherm RHTH tube oven with a maximum of 1800 °C heating capacity at 2 mbar pressure. All the *ex situ* experiments reported in this article were performed at 400 and 450 °C for 10 min, after carrying out a few pilot experiments at 350, 400, and 500 °C to optimize the growth temperature. The samples for *ex situ* vacuum oven growth were prepared similarly as they were made for *in situ* TEM heating.

## Results and Discussion

### Thermal Decomposition of Ammonium Metavanadate

A single solid-state precursor, ammonium metavanadate (NH_4_VO_3_, abbreviated as AMV), was heated in the *in situ* TEM experiments to observe and investigate the growth of crystalline vanadium oxide structures, V_2_O_5_ and VO_2_, using a dedicated *in situ* heating holder. The previous research reported on the thermal decomposition of AMV in different gaseous environments (vacuum, argon, nitrogen, and air) to produce V_2_O_5_, which can be shown with the following simple equation:^29−32^
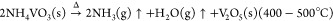
1

The reaction is much more complicated than shown in the above equation where different intermediate products at intermediate temperatures can be obtained, but these are mostly very poorly crystalline or even amorphous.^29−32^ This suggests that thermal decomposition of the AMV precursor leads to the growth of the only pure crystalline structure, V_2_O_5_. This is one of the easiest and less complex methods to grow crystalline V_2_O_5_ structures, which can be used for various applications. Apart from one of the *in situ* TEM experiments where amorphous V_2_O_5_ was transferred to crystalline orthorhombic V_2_O_5_,^58^ not many attempts were made to observe the growth of vanadium oxide-based materials via *in situ* TEM. The V_2_O_5_ nanobelts^59^ and nanosheets^28,60,61^ prepared with this method of thermal decomposition of AMV precursor show to be an excellent candidate as a cathode for Li-ion battery applications in comparison to the conventionally grown nanobelts and nanosheets using amorphous or crystal V_2_O_5_ precursors.^17,19,20,62,63^ Although precise control over the thickness and growth of the nanobelts or nanosheets is not a strong feature of this method, it provides a great opportunity to observe and investigate the growth of V_2_O_5_-based structures through *in situ* TEM experiments with no requirements of specialized instruments. Besides this, the synthesis route can be likely scaled up and fine-tuned with the help of the *in situ* TEM findings.

### *In Situ* TEM Growth of V_2_O_5_ Nanostructures

An AMV precursor in ethanol solution was drop cast onto the ∼30 nm thick SiN_*x*_ membrane of a TEM heating chip and dried in air to prepare samples for *in situ* TEM experiments. With the help of a controlled heating setup, the prepared heating chip was introduced into the TEM column. The schematics in Figure S1 depict the whole scenario of preparing a heating chip for the *in situ* TEM experiments. To remove any residues present in the solvent on the heating chip, it was heated from room temperature to 100 °C for ∼10 min (see Figure S2 and Supporting Note 1 in the Supporting Information for more details). The temperature was then increased to 700 °C in steps of 20 °C withholding the temperature at different stages for a few minutes to monitor and capture the frames of the changes in the precursor.

**Figure 1:**
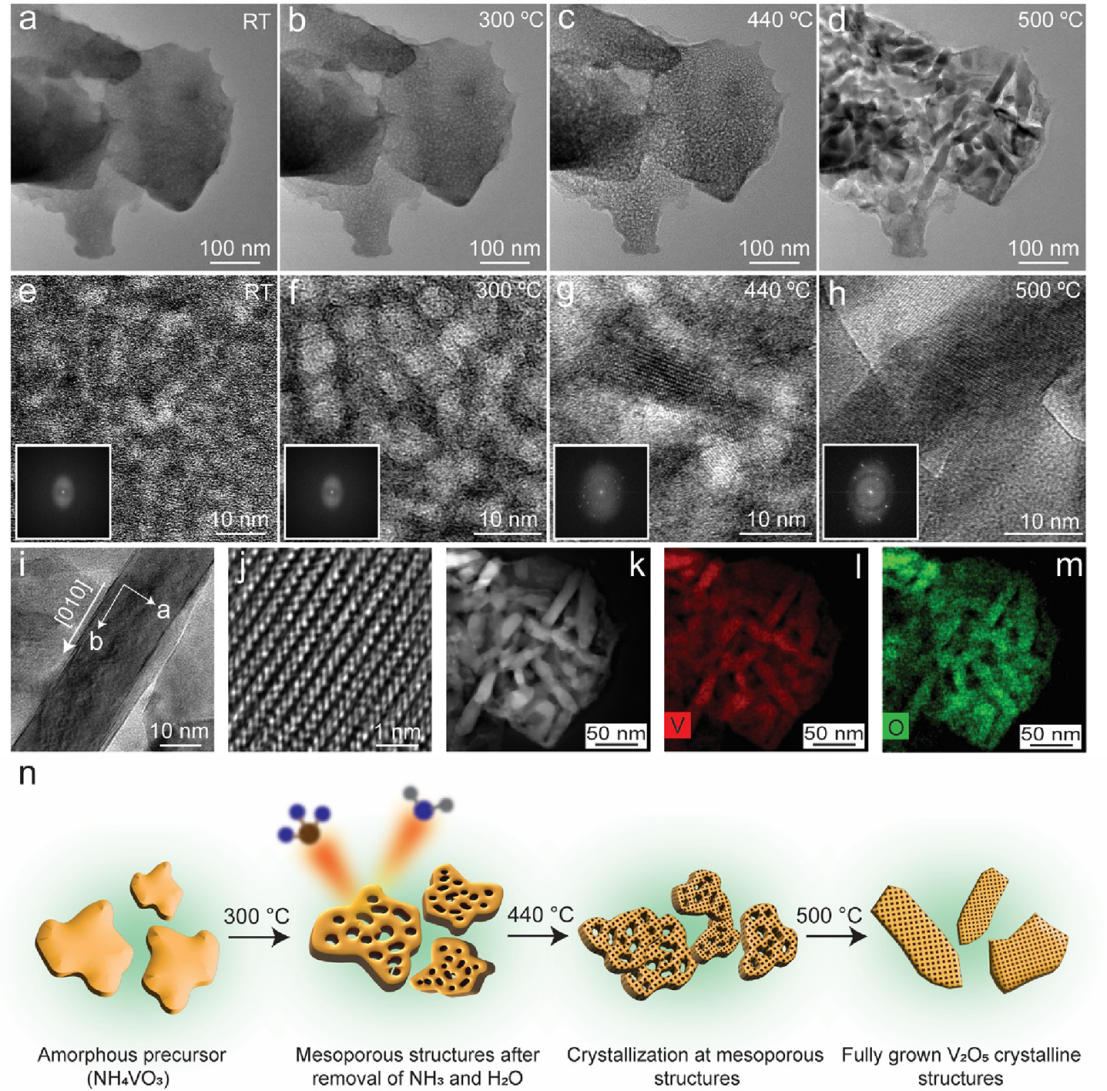
Growth of crystalline structures of V_2_O_5_ during *in situ* TEM heating of AMV precursor. (a–d) Low-magnification TEM images showing: (a) an amorphous AMV precursor at room temperature (RT) as drop cast onto the heating chip. (b) A mesoporous structure formed at 300 °C. (c) Evolution of crystalline structures on mesoporous structures at around 440 °C. (d) Fully grown crystalline V_2_O_5_ structures at 500 °C. (e–h) High-magnification TEM images showing: (e) AMV precursor at RT with a diffuse ring in the fast Fourier transform (FFT) in the inset. (f) Formation of holes in the precursor at 300 °C and a diffuse ring in the FFT in the inset. (g) Initiation of crystallization in the mesoporous structure at 440 °C, evident from the appearance of dots in the FFT in the inset. (h) One of the fully grown V_2_O_5_ structures with lattice fringes. The corresponding FFT in the inset shows an increased number of dots. (i) The TEM image of one of the V_2_O_5_ nanobelts (NB) grown out of AMV precursor. (j) The high-resolution TEM image of the nanobelt showing atomic columns. (k) The HAADF-STEM image of the V_2_O_5_ structures from panel d. (l, m) STEM-EDS elemental maps for vanadium (V) and oxygen (O) in V_2_O_5_ structures. (n) Scheme presenting the overall process of thermal decomposition of AMV precursor to fully grown V_2_O_5_ structures with temperature.

A brief story of the growth of crystalline V_2_O_5_ structures through thermal decomposition of a single, solid-state, and amorphous AMV precursor with *in situ* TEM heating is shown in Figure 1. The amorphous AMV precursor at room temperature (RT) in TEM gives an image as shown in Figure 1a. Figure 1e shows a close-up look at the amorphous AMV precursor along with the diffuse ring in the fast Fourier transform (FFT) shown in the inset, from which it is evident that the precursor is amorphous prior to any heat treatment. With the temperature reaching 300 °C in Figure 1b, the amorphous precursor starts to form a mesoporous structure by forming holes that are clearer in Figure 1f and the structure remains amorphous as is evident from the absence of any bright dots in the FFT in the inset in Figure 1f. The first appearance of the crystalline structures on the mesoporous amorphous structure was captured at a temperature of 440 °C. The high-magnification TEM image along with the presence of dots in the FFT in the inset in Figure 1g manifests the amorphous-to-crystalline transition at 440 °C. Figure 1d shows the emergence of well-defined shapes of nanobelts (NBs) of crystalline V_2_O_5_, which becomes clearer in Figure 1h and with the larger number of bright spots in FFT in the inset. The V_2_O_5_ NBs are seen to be grown along the *b* axis as shown in Figure 1i. A close inspection at the edges of the V_2_O_5_ NB in Figure 1i suggests that it has a multilayer configuration, which is typical of the structure of an NB. Figure 1j shows the high-resolution TEM (HRTEM) image from the part of the V_2_O_5_ NB in Figure 1i. This shows the typical arrangements of atomic lattices in orthorhombic V_2_O_5_ structures.

A slightly zoomed-in HAADF-STEM image of the grown V_2_O_5_ structures at 500 °C, along with the STEM-EDS maps for vanadium and oxygen, is shown in Figure 1k–m, respectively. The schematic in Figure 1n represents the overall process of the growth of crystalline V_2_O_5_ structures from amorphous precursor with increasing temperatures. The mesoporous structure forms after heating the precursor to 300 °C after the removal of NH_3_ and H_2_O as mentioned in eq 1. This becomes clearer once the crystallization of V_2_O_5_ starts after heating to 440 °C as evidenced by the difference in contrast as seen in Figure 1c. Increasing the temperature further to 500 °C forms fully grown, nicely defined, and crystallized V_2_O_5_ structures.

**Figure 2:**
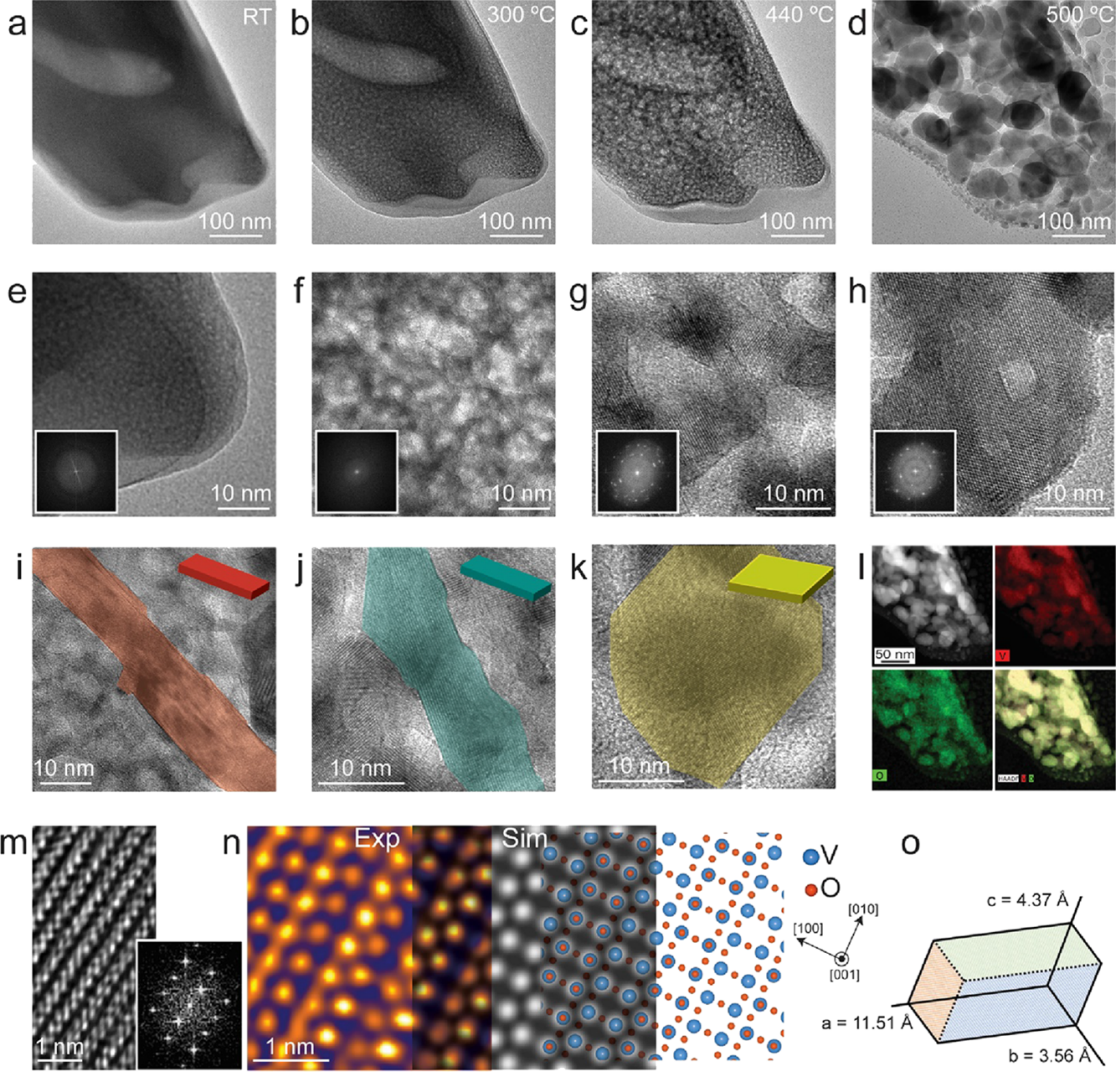
*In situ* growth of distinct V_2_O_5_ structures. (a–d) TEM images were captured at: (a) room temperature with an amorphous AMV precursor, (b) 300 °C with a mesoporous amorphous precursor, (c) 440 °C with initialization of crystallization of V_2_O_5_, and (d) 500 °C with fully grown structures of V_2_O_5_. (e–h) Magnified TEM images along with the corresponding FFTs in the inset for panels a–d, respectively. (i, j) Few-layered nanobelt structures of V_2_O_5_ with growth dominant along one direction. (k) Growth in two directions of the nanosheet (NS)-like structures of V_2_O_5_. The colors overlayed on the structures are used to emphasize the observed 1D NB and 2D NS structures. (l) The HAADF-STEM image and EDS maps for V and O along with the altogether overlayed image from the area shown in panel d. (m) The spatially resolved HRTEM image from one of the fully grown V_2_O_5_ structures shown in panel h along with the corresponding FFT. (n) Combination of overlapped experimental, simulated HRTEM images, and an atomic model for orthorhombic V_2_O_5_ projected along the (001) direction (the TEM image was processed with the GEM LUT effect in ImageJ software for better visualization). The blue and red balls represent vanadium (V) and oxygen (O) atoms, respectively. The orthographic projection shows the viewed direction. (o) The sketch of an orthorhombic unit cell of V_2_O_5_.

The *in situ* TEM observations suggest that there are different types in the final morphology of the V_2_O_5_ structures, which can be subdivided into 1D NBs and 2D nanosheets (NSs). Figure 1 shows the dominant growth of few-layered 1D NBs of V_2_O_5_, whereas Figure 2 shows the growth of V_2_O_5_ structures progressed along 2D, which are designated as NSs. Figure 2 shows the gradual growth of crystalline V_2_O_5_ structures in a completely independent experiment, at the same temperatures shown in Figure 1 but with a major difference in the final morphology. The blurred TEM image in Figure 2a at RT along with the corresponding FFT in the inset of Figure 2e confirms the amorphous nature of the AMV precursor. Figure 2b,f confirms the formation of the porous structure of the precursor at 300 °C after the removal of NH_3_ and H_2_O molecules as mentioned in an earlier section. Crystallization of V_2_O_5_ starts to occur at 440 °C, which is evident from the clear appearance of lattice fringes in Figure 2g and the increased number of bright spots in the FFT in the inset of Figure 2g, which gives the contrast difference in Figure 2c. Figure 2d shows fully grown crystalline V_2_O_5_ structures at 500 °C, which is different from the structures (NBs) seen in Figure 1d. One of the nicely crystalline structures is shown in Figure 2h, which gives an impression of a few-layered 2D nanosheet. Figure 2i,j shows an image of typical NBs observed at a different location on the heating chip at the temperature of 500 °C. The colors overlayed in Figure 2i,j on the V_2_O_5_ structures (red and dark cyan, respectively), along with the schematic models, suggest the one-directional growth of NBs, whereas the golden yellow color overlayed on the V_2_O_5_ structure, and the schematic model in Figure 2k, proposes the 2D growth of NSs. Figure 2l depicts the HAADF-STEM image and EDS maps for vanadium and oxygen for the same area as shown in Figure 2d, which also helps to confirm the observed structures of V_2_O_5_. A typical atomic column image for orthorhombic V_2_O_5_ along with the FFT is shown in Figure 2m. A filtered TEM image of the atomic columns in V_2_O_5_ is matched with the simulated image and atomic model for orthorhombic V_2_O_5_ in Figure 2n. This suggests that the observed image is visualized along the *c* axis. Figure 2o shows the sketch of an orthorhombic unit cell of V_2_O_5_. The highly resolved atomic column images of V_2_O_5_ are viewed through the *c* axis direction of the unit cell. Most of the NBs and NSs are oriented in the [001] zone axis, which is the basal plane, with a few of them oriented in other directions as well. However, we specifically choose to monitor those that are oriented in the [001] zone axis to obtain better images and hence obtain a better understanding of the crystal structure.

It was found that the electron beam did not have a significant influence on the observations, which became clear from reference measurements (see Supporting Note 2 and Supporting Figure S3): after keeping one of the precursor areas on the heating chip under the exposure of the electron beam at room temperature for more than 10 min, it did not lead to any significant changes in it, as evidenced by the absence of any visible lattice fringes in the TEM images in Figure S3a, b. A similar process of the thermal decomposition of AMV precursor as observed in the *in situ* heating with continuous exposure of precursor to the electron beam is observed when the precursor was kept away from the electron beam by blanking it during the entire period of heating (see Figure S3c–f and Supporting Note 2).

Several *in situ* TEM experiments were carried out in which both these structures of V_2_O_5_ (NBs and NSs) are observed on the same heating chip and at the same locations in mixed forms. This can be the result of multiple factors, out of which the thickness of the AMV precursor before heating could be one. In our previous work on WS_2_ growth through thermolysis, we observed the selective growth of vertical and horizontal WS_2_ flakes depending on the thickness of the precursor.^54^ In the present study, however, controlling the thickness of AMV precursor was not a straightforward task, and besides that, both structures are observed to grow in a mixture without a clear clue on how to gain control over the one type of structure. This keeps an interesting question open for a further scientific explanation to obtain the desired type of growth of V_2_O_5_ through this clean method. Because of the random mixture of these morphologies, it is difficult to specify the distribution of them within the sample, but for all of the experiments, the appearance of growth of NBs and NSs is similar.

**Figure 3:**
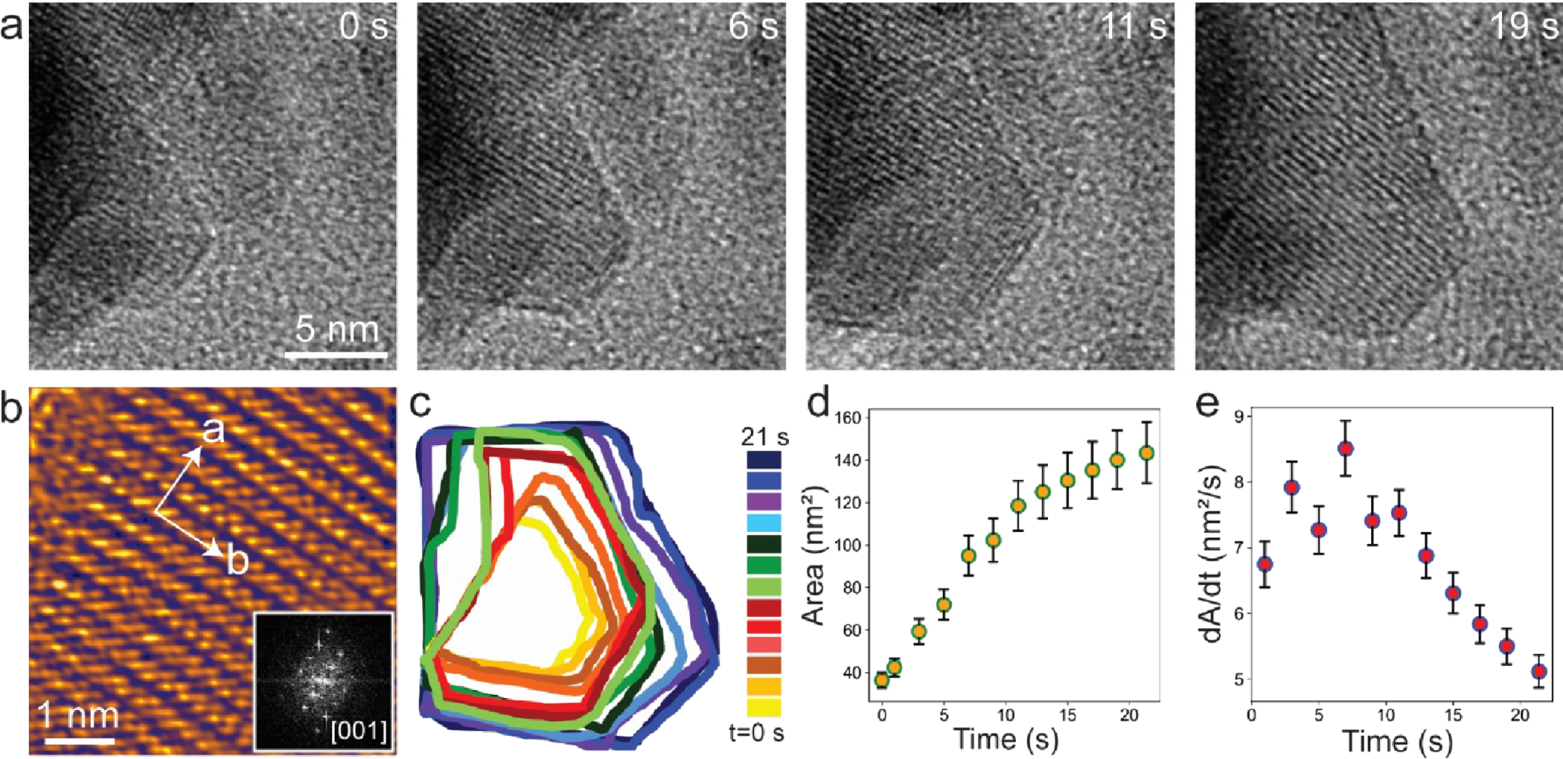
Real-time observation of growth of the V_2_O_5_ nanosheet. (a) Time-series frames at different growth stages. (b) The high-magnified filtered image from the grown V_2_O_5_ NS and FFT in the inset (the TEM image was processed with the GEM LUT effect in ImageJ software for better visualization). (c) Contour maps at different growth steps. (d, e) Plots of the increased area of NS with time and the growth rate at different times, respectively.

Real-time observations of the growth of nanostructured materials provide interesting and vital information with the help of which understanding of and control over the growth process can be gained much more efficiently.^48,50−54,56^ The time-series frames captured at different growth stages for V_2_O_5_ NSs are presented in Figure 3a. The movie (Supporting Movie1) was recorded at the midway temperature of 460 °C after the crystalline V_2_O_5_ grows out of an amorphous precursor. Movie frames recorded after 0, 6, 11, and 19 s are displayed in Figure 3a and show the gradual in-plane growth of the NS in two directions. The zeroth second in the first frame in Figure 3a is indexed to show the beginning of the growth of NS, and the following times refer to the different growth stages. It can be seen from the frames in Figure 3a that the NS grows with sharp edges and corners with good crystallinity. Figure 3b shows a filtered image from the last frame of the movie, which displays atomic columns in the orthorhombic V_2_O_5_ NS that is oriented horizontally parallel to the SiN_*x*_ membrane of the heating chip. In all of the *in situ* experiments, it has been observed that almost all of the V_2_O_5_ structures are lying flat on the SiN_*x*_ membrane with a *c* axis parallel to the viewing direction. This is evident from all of the figures (Figures 1, 2, and 3) discussed in earlier sections. The FFT in the inset of Figure 3b displays the single-crystalline nature of the grown orthorhombic V_2_O_5_ NS. The multicolored contour maps in Figure 3c showing the time evolution give an idea about the average growth of the V_2_O_5_ structures. This show that, during the growth, the area of the NS initially increases fast, while it grows relatively slowly further on as shown in the plot in Figure 3d. The growth rate plot in Figure 3e shows that it increases sharply for a few seconds and then drops down gradually until the twentieth second when the NS does not show any further growth. This in turn explains the limited area growth of NBs and NSs displayed in Figures 1 and 2.

Movies of NB growth are not included as these were found to grow too fast after changing the temperature to the growth temperature. We attempted to capture the real-time growth process of NBs but these either grew fast with the growth temperature, or did not transform into a clear shape when continuously imaged with the electron beam. This could be the effect of the electron beam on the growth, an effect that was not observed during the growth of NSs. *In situ* movies of NB growth are therefore not included in order not to report results that may be the result of the electron beam effects.

### Phase Transition of 2D V_2_O_5_ to 3D VO_2_ Nanostructures

Obtaining two different materials of the same elements from one precursor can be an efficient way of synthesis. Phase transitions in materials provide the capability to produce two differently characterized materials that can be used in multiple applications individually. As discussed in earlier sections, the amorphous AMV precursor transforms into various crystalline V_2_O_5_ nanostructures. It has been observed previously that the V_2_O_5_ can be transformed into multiple phases of VO_*x*_.^64−67^

**Figure 4:**
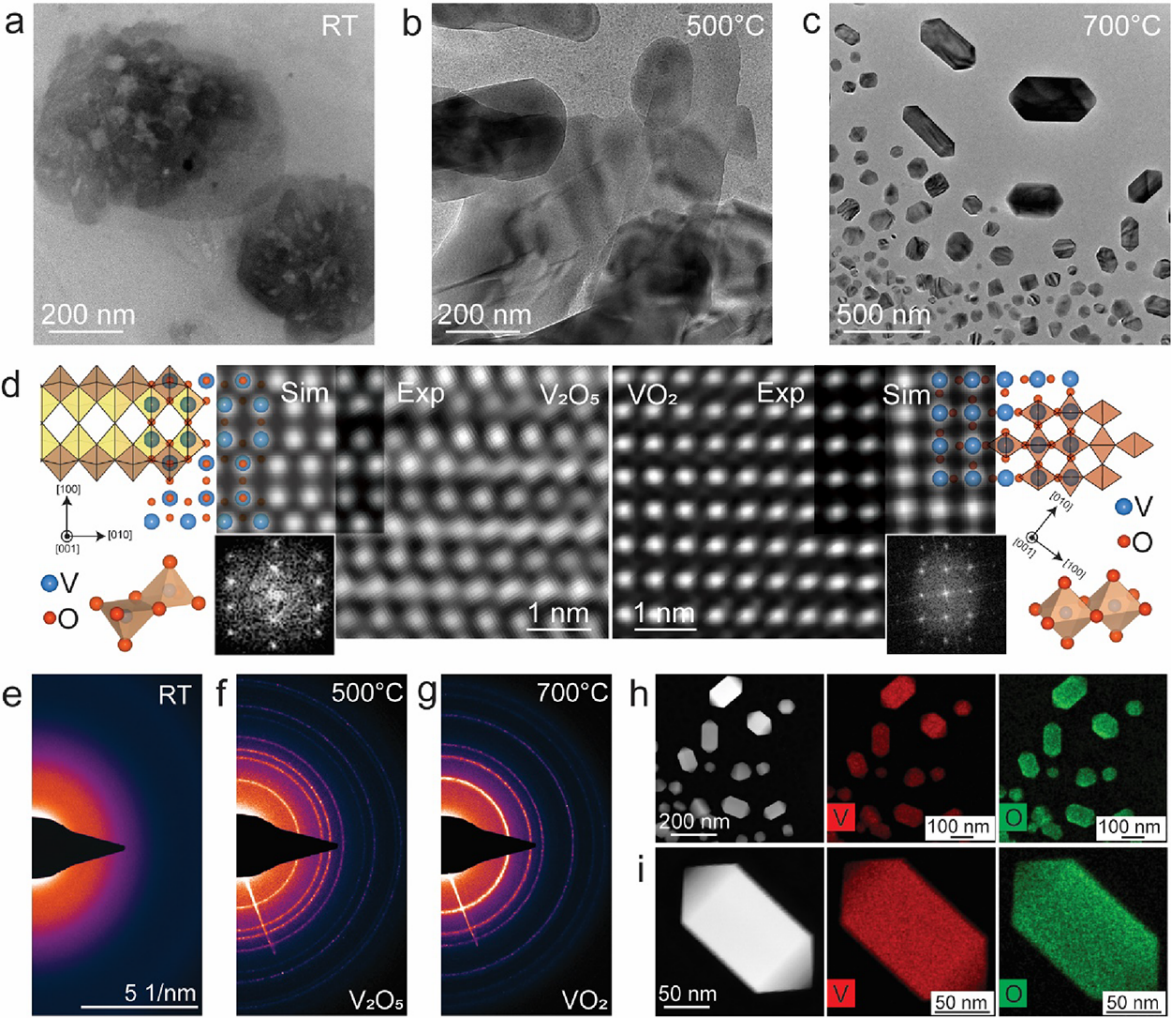
Phase transition in V_2_O_5_. (a) An amorphous AMV precursor at RT. (b) A few layered crystalline V_2_O_5_ structures at 500 °C. (c) Nanostructures of VO_2_ at 700 °C. (d) Combination of experimental TEM images mapped with simulated images and atomic models for both V_2_O_5_ and VO_2_ along with the corresponding FFTs. The blue and red balls in both cases represent vanadium and oxygen atoms, respectively. (e–g) Electron diffraction patterns of AMV precursor, crystalline V_2_O_5_, and VO_2_ at the corresponding temperatures. (h) The HAADF-STEM image and EDS elemental maps for vanadium and oxygen of multiple and (i) single VO_2_ structures after heating to 700 °C.

Figure 4a shows the amorphous AMV precursor at room temperature, which then transformed into the thin-crystalline V_2_O_5_ NBs and NSs at 500 °C as shown in Figure 4b. When increasing temperature further, the V_2_O_5_ nanostructures show good thermal stability until the temperature reaches 700 °C. At that temperature, the thin nanostructures of V_2_O_5_ collapse into sharp-edged three-dimensional nanoparticles, which is evident from their shape as displayed in Figure 4c. These nanoparticles show various shapes and sizes along with different thicknesses, but their three-dimensional and strongly faceted nature is a common factor, unlike thin V_2_O_5_ nanostructures. The crystallinity difference of the V_2_O_5_ nanostructures from the obtained nanoparticles is shown in Figure 4d with the help of filtered HR-TEM images, simulated images, schematics of atomic models, and FFTs. The EDS elemental maps in Figure 4h,i for vanadium and oxygen from a group of nanoparticles, and of the larger individual sharp-edged nanoparticle, show a 1:2 stoichiometry between V and O, which confirms the VO_2_ composition. The process of the phase transition from V_2_O_5_ to VO_2_ was so rapid that capturing it through TEM was impossible, which is why there are no videos capturing this. Increasing the temperature to 700 °C made the V_2_O_5_ structures transform no time into particles of VO_2_.

A detailed analysis of the atomically resolved images in Figure 4d of the three-dimensional nanoparticles gives interplanar distances of 0.45 nm, which represents the (010) plane for monoclinic, and (100) and (010) planes for rutile, phases of VO_2_.^68^ The insulator-to-metal transition between the two phases of VO_2_ occurs near room temperature in which the monoclinic phase is the low-temperature phase, while rutile is the high-temperature phase.^69,70^ Thus, it suggests that the nanoparticles obtained after the phase transition from V_2_O_5_ at 700 °C show a rutile phase of VO_2_ as it stays in this phase without showing any changes after heating to 740 °C. During the experiments, the monoclinic phase may have been formed as an intermediate phase as well while this was not recorded in the images as the temperature is quite high, or the rutile phase was formed directly not via the formation of the monoclinic phase. Increasing the temperature to 740 °C does not change the crystal structure, and this also suggests that the observed phase is rutile, which is stable at higher temperatures. As both these phases, monoclinic and rutile, have different properties (insulating and metallic), they have different applications based on their properties. In our experiments, we found that the rutile phase of VO_2_ is stable at 740 °C, and therefore, it is possible to form this as a stable nanoscale phase under controlled experimental conditions. The VO_2_ particles that converted from the NBs and NSs of V_2_O_5_ were found oriented in random directions as they can be seen in 3D shapes in contrast to the 2D NBs and NSs that were all found in (nearly) the same orientation. The temperature profile plot in Figure S2 shows the increased temperature from 700 to 760 °C with the same phase of VO_2_. In a separate experiment, the AMV precursor was heated inside the microscope and the electron diffraction patterns (DPs) of the phase transition were recorded and are presented in Figure 4e–g. Figure 4e shows the blurred DP without any sharp rings at room temperature corresponding to the amorphous AMV precursor. At 500 °C, the phase transition occurred from amorphous AMV precursor to crystalline V_2_O_5_, which is evident from the bright rings in the DP in Figure 4f. Further increasing the temperature to 700 °C leads to the structural transition from orthorhombic V_2_O_5_ to rutile VO_2_, and the corresponding bright rings in the DP are evident of this transition. The details on the DPs with indexing of the bright rings to the corresponding planes in the V_2_O_5_ and VO_2_ are shown in Supporting Figure S4.

### *Ex Situ* Growth of V_2_O_5_ and VO_2_ Nanostructures

**Figure 5:**
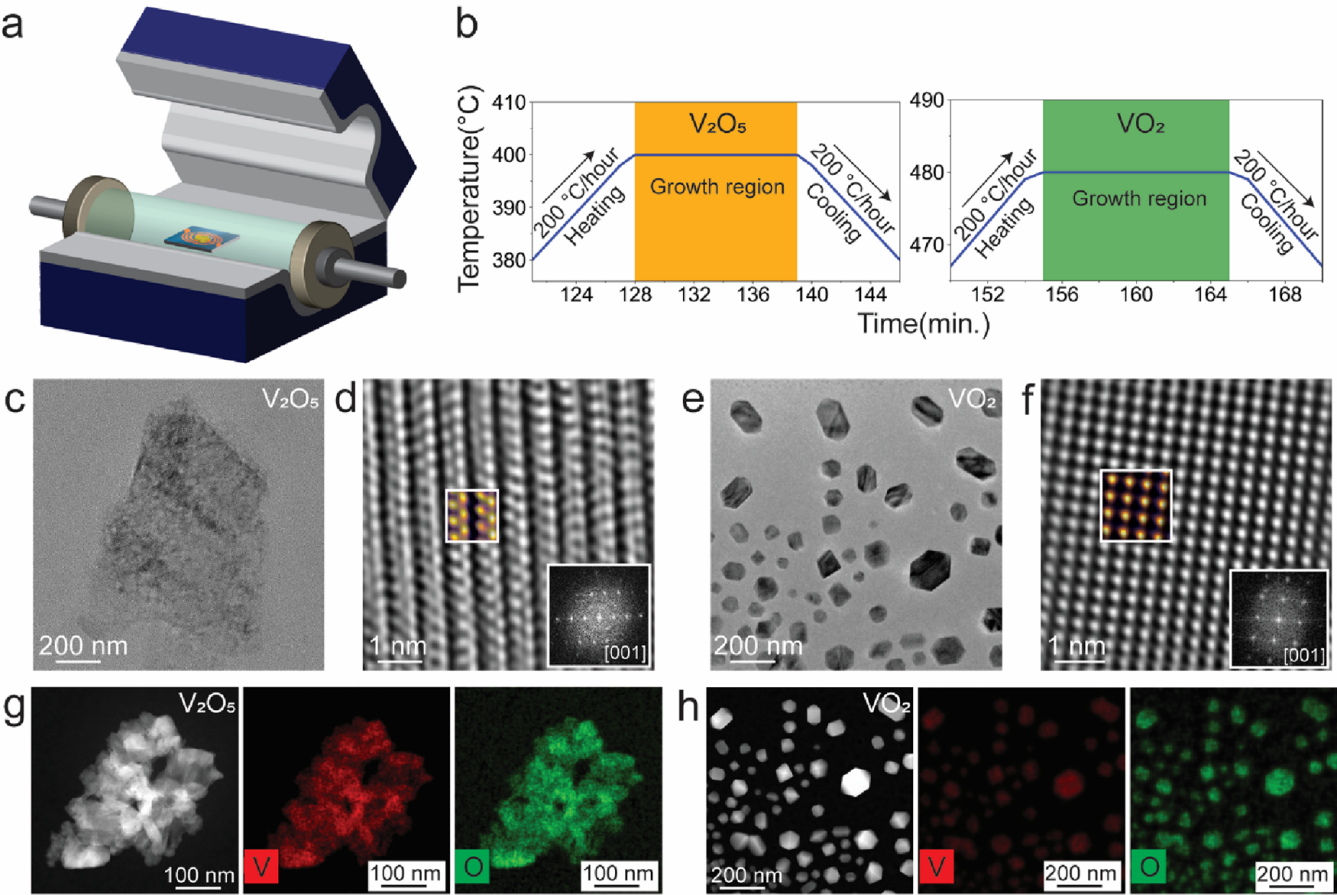
*Ex situ* growth of vanadium oxide structures. (a) Schematic representation of the setup for the *ex situ* experiment. (b) Temperature profiles for both V_2_O_5_ and VO_2_ structures. (c) The TEM image of a thin V_2_O_5_ nanosheet. (d) The high-resolution image of orthorhombic V_2_O_5_ with an overlapped colored simulated image in a box, and the FFT in the inset. (e) The TEM image of VO_2_ nanostructures. (f) The high-resolution TEM image of rutile VO_2_ with an overlapped colored simulated image in a box, and FFT in the inset. (g, h) HAADF-STEM images of V_2_O_5_ and VO_2_ structures in the first panels and EDS elemental maps for vanadium and oxygen in red and green colors, respectively.

An *in situ* TEM experiment provides important information on optimization and control of the growth of materials along with insights into the evolution of the growth. To scale up the synthesis or fabrication of the materials, it is necessary to grow materials in regular laboratory conditions. Hence, to support the *in situ* findings in thermolysis-driven growth of vanadium oxide nanostructures, *ex situ* experiments were carried out in the vacuum oven as depicted in the schematic in Figure 5a. The heating chips drop cast with AMV precursor were kept in the central part of the vacuum oven after setting it for the temperature controls. Here, in the *ex situ* experiments, the same heating chips are used to keep the same substrate interaction with the precursor and to observe the grown structures directly in the microscope without carrying any distinct sample preparation method. This gave a feasible opportunity to observe the as-grown structures without any possible disturbance from the electron beam. The temperature profiles during the growth for both V_2_O_5_ and VO_2_ are shown in Figure 5b. In both cases, the heating and cooling rates were kept the same at 200 °C/h, and the temperature platforms of 400 and 480 °C for the growth of V_2_O_5_ and VO_2_ were kept for time periods of 10 and 12 min, respectively. The growth temperatures for both V_2_O_5_ and VO_2_ were optimized by carrying out several pilot experiments. One of the examples of the *ex situ* grown thin nanosheets of V_2_O_5_ is shown in Figure 5c, and the high-resolution image with the colored simulated image in the indicated box, along with the FFT pattern, is shown in Figure 5d. The typical arrangements of atomic columns in the orthorhombic V_2_O_5_ are visible in the experimental high-resolution image and in the simulated image under the same conditions as that of the *in situ* experiment. The same sample transforms to the VO_2_ after being heated to 480 °C, and the nanostructures of VO_2_ are shown in Figure 5e along with the high-resolution image from one of the nanostructures in Figure 5f, with a colored simulated image in the indicated box, and the FFT pattern in the inset. HAADF-STEM images show the thinness of the V_2_O_5_ NSs in Figure 5g along with the elemental maps for V and O in red and green colors, respectively. The thin nanosheets of V_2_O_5_ transformed into the thick nanoparticles of VO_2_, which is evident from the HAADF-STEM image in Figure 5h, where the shape of the particles is seen to be different from that of the thin nanosheets. The EDS elemental maps for V and O are represented in red and green, respectively, and the quantification of the EDS information shows a stoichiometry of 1:2 in VO_2_. Thus, overall, the *ex situ* experiments performed in the vacuum oven produced similar structures of V_2_O_5_ and VO_2_ nanostructures at slightly different temperatures compared to those in the *in situ* TEM experiments. The pressure difference between the TEM column and vacuum oven can be a major factor causing the transformation or other thermodynamic processes to take place at quite different temperatures. Also, in the current case, the pressure difference in the TEM column and vacuum oven plays a role, and hence, the phase transformation of V_2_O_5_ to VO_2_ occurred at 480 °C in the *ex situ* experiment. Another aspect is the local nature of the heating inside the *in situ* TEM heating setup vs the broad *ex situ* heating inside the vacuum oven, which might also have played a role resulting in differences in the observed temperatures for phase transformations.

## Conclusions

In summary, we observed thermolysis-driven growth of V_2_O_5_ nanostructures on SiN_*x*_ membrane using a single solid-state precursor in combination with *in situ* TEM heating. Intermediate stages during the thermolysis of AMV precursor are observed in real time, which would be unreachable when using conventional methods. The growth temperatures required in the thermolysis of the AMV precursor to produce fully grown V_2_O_5_ nanostructures are optimized. The phase transformations from V_2_O_5_ to VO_2_ are also observed in the *in situ* TEM experiments at elevated temperatures. Furthermore, the *ex situ* thermolysis of AMV precursor in a vacuum oven produced pure crystalline V_2_O_5_ and VO_2_ nanostructures on the bare SiN_*x*_ membrane of the heating chip, which validates the thermolysis-driven growth as observed by means of *in situ* TEM. Controlling the thickness of the precursor and hence the thickness of grown 2D V_2_O_5_ nanostructures, along with the optimization of the parameters to control the morphology of V_2_O_5_ nanostructures, are still open challenges of this method. Our study offers a relatively simple method to produce pure crystalline V_2_O_5_ 1D nanobelts and 2D nanosheets along with VO_2_ nanostructures in a single-synthesis method that can be scaled up to produce desired materials for rechargeable batteries and catalytic applications.

## Supporting Information Available

Additional figures show a schematic illustrating the drop-casting of ATT onto the heating chip and examination using the electron beam after the introduction of it in the TEM column for conducting the *in situ* TEM experiment, *in situ* heating temperature profiles of experiments describing the observed growth of different structures at particular temperature ranges, TEM images of the AMV precursor kept under the electron beam for more than 10 min at room temperature before and after the exposure, electron diffraction patterns (DP) during the *in situ* TEM heating experiment for the amorphous precursor, and orthorhombic V_2_O_5_, and rutile phase VO_2_ structures (PDF)
Growth of the V_2_O_5_ layer at 450 °C (slowed down to 1/2 times of real time) (Supporting Movie 1) (AVI)

## Supplementary Material

an3c00397_si_001.pdf

an3c00397_si_002.avi
