# Supplementary material for: Thermolysis-Driven Growth of Vanadium Oxide Nanostructures Revealed by In Situ Transmission Electron Microscopy: Implications for Battery Applications
Source: ACS Appl Nano Mater. 2023 May 3;6(9):7280–9. doi: 10.1021/acsanm.3c00397 (PMC10186331; doi:10.1021/acsanm.3c00397)
Supplement: Supplementary file 1 — an3c00397_si_001.pdf [file an3c00397_si_001.pdf]

Supporting Information

Thermolysis Driven Growth of Vanadium Oxide Nanostructures Revealed by *In Situ* Transmission Electron Microscopy: Implications for Battery Applications

*Dnyaneshwar S. Gavhane, Atul D. Sontakke, Marijn A. van Huis**

Dnyaneshwar S. Gavhane, Dr. Marijn A. van Huis
Soft Condensed Matter,
Debye Institute for Nanomaterials Science,
Utrecht University,
Princetonplein 5, 3584 CC, Utrecht,
The Netherlands

Atul D. Sontakke
Condensed Matter and Interfaces,
Debye Institute for Nanomaterials Science,
Utrecht University,
Princetonplein 5, 3584 CC, Utrecht,
The Netherlands

Corresponding author

*Email: m.a.vanhuis@uu.nl

Supplementary Text

Supporting Note 1:

The *in situ* TEM heating experiments were carried out several times (more than 10 times) to consolidate findings of the growth in each experiment, under the same experimental conditions. As seen from Figure S2, the average time for the number of experiments was about 3 hours. In many experiments, the AMV precursor was heated to 100 °C for 10 mins (flat lines in the plots at 100 °C) to get rid of any residues from the solvent. The area shaded with light pink (~440 °C) in the profile, represents the initiation of crystallization of V_2O_5 structures. A light orange highlighted regime at ~500 °C indicates a region where the well-defined crystallized structures of V_2O_5 occurred. The blue-highlighted region indicates the thermal stability of V_2O_5 structures for a longer time at ~560 °C. The area marked with a purple patch shows the transformation of orthorhombic V_2O_5 structures into the rutile phase VO_2 structures at 700 °C. Upon further heating at temperatures > 750 °C, VO_2 is transformed into elemental metallic V islands, and this region is yellow-highlighted in Figure S2. A few experiments were done at high heating rates to observe the possible effects of rapid heating on growth. Higher heating rates did not lead to any differences in the structures of the grown structures of V_2O_5. The structures grow at particular growth temperatures also when rapid heating was applied.

Supporting Note 2:

The AMV precursor area shown in Figure S3 was brought under the electron beam only to capture the images at room temperature, 300 °C and 440 °C. To rule out any effects of the electron beam on the growth of nanostructures or local heating because of electron beam illumination the area shown in Figure S3 was intentionally kept away from the electron beam by blanking the beam during the entire period of heating. As the similar growth of nanostructures in the non-illuminated precursor area is observed and is shown in Figure S3(e & f), the results suggest negligible effects of the electron beam on the growth of nanostructures. This validates the fact that the growth of V_2O_5 nanostructures is global and uninfluenced by the electron beam.

Supporting Figures

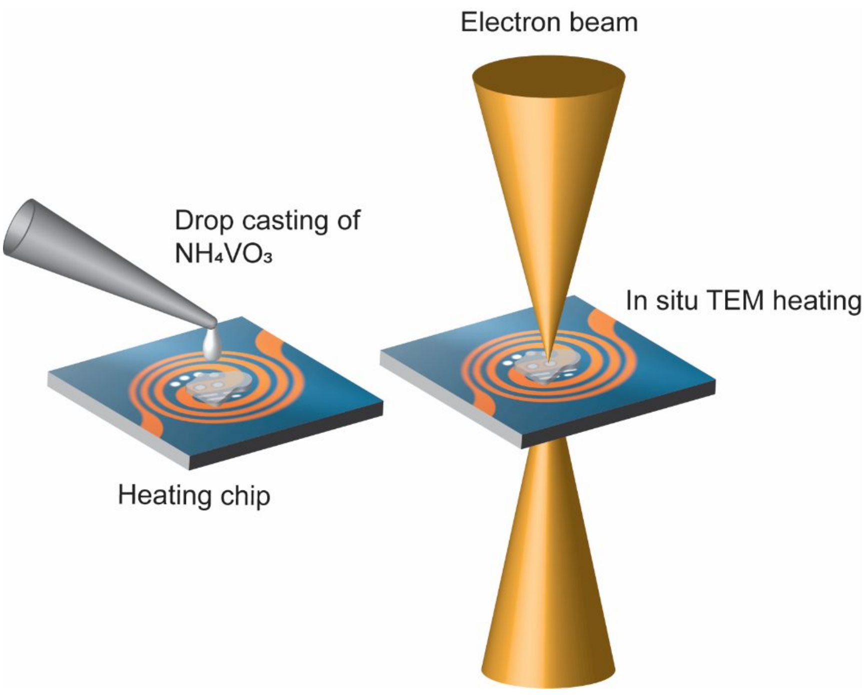

Figure S1. Schematic illustrating the drop-casting of ATT onto the heating chip and examination using the electron beam after the introduction of it in the TEM column for conducting the *in situ* TEM experiment.

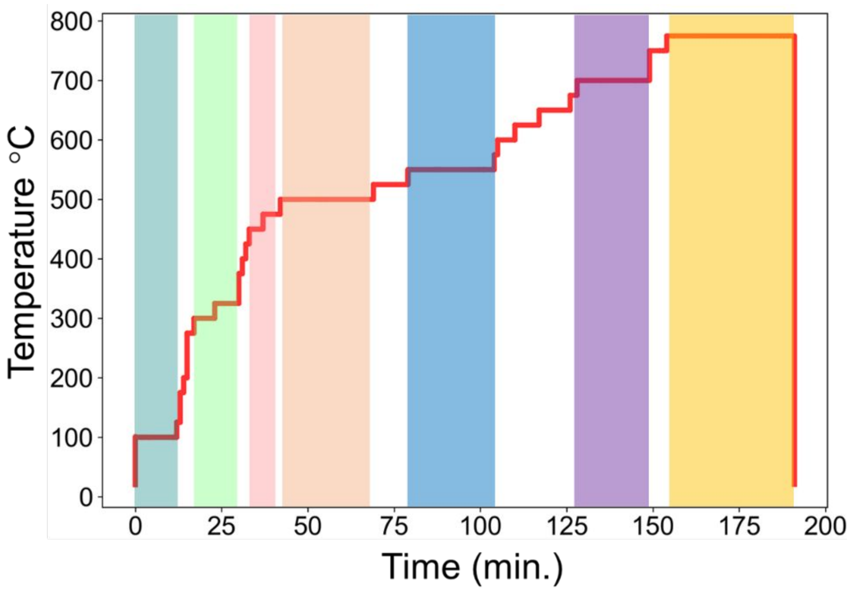

Figure S2. *In situ* heating temperature profiles of experiments describing the observed growth of different structures at particular temperature ranges.

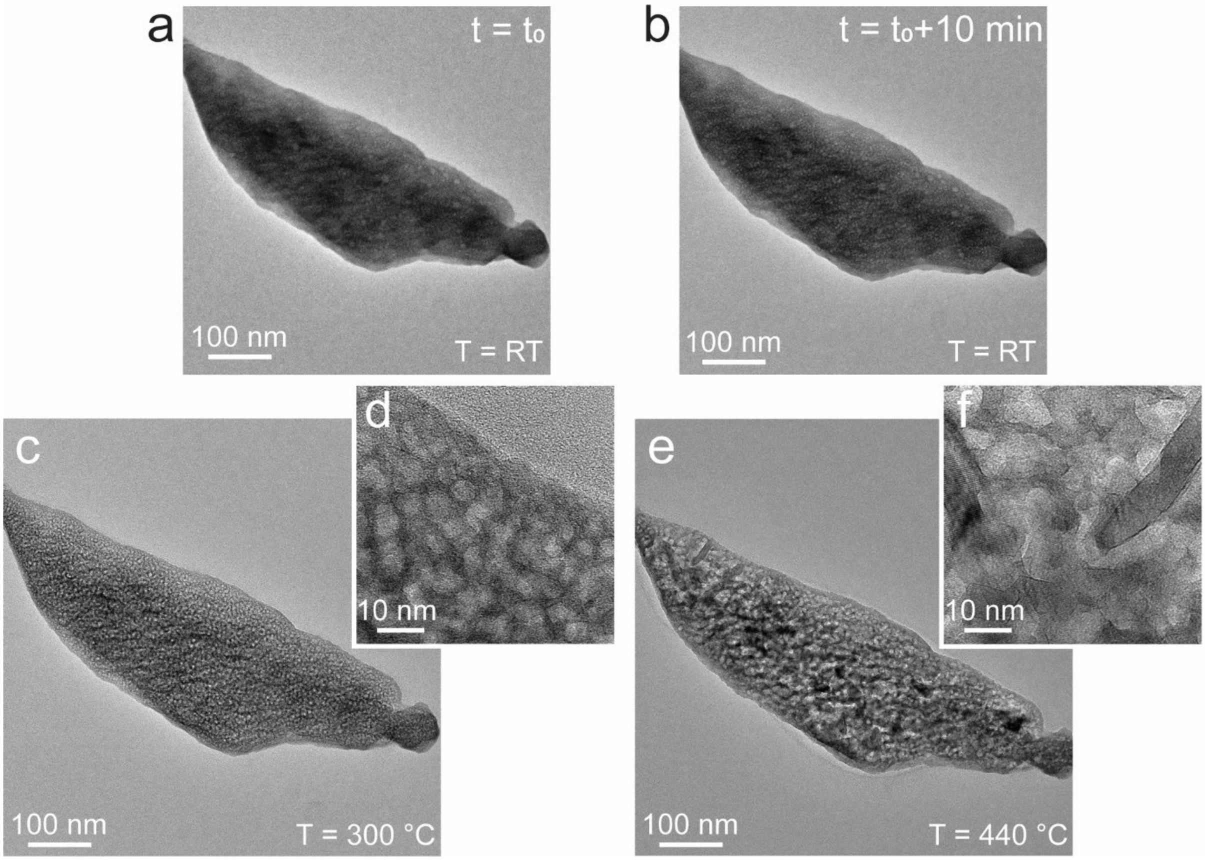

Figure S3. The AMV precursor was kept under the electron beam for more than 10 minutes at room temperature (a) before and (b)after exposure, not resulting in the growth of any crystalline V_2O_5 nanostructures. (c & d) Images captured at 300 °C show the porous structure at the precursor. (e & f) Images captured at 440 °C show the beginning of crystallization with few already grown crystalline structures.

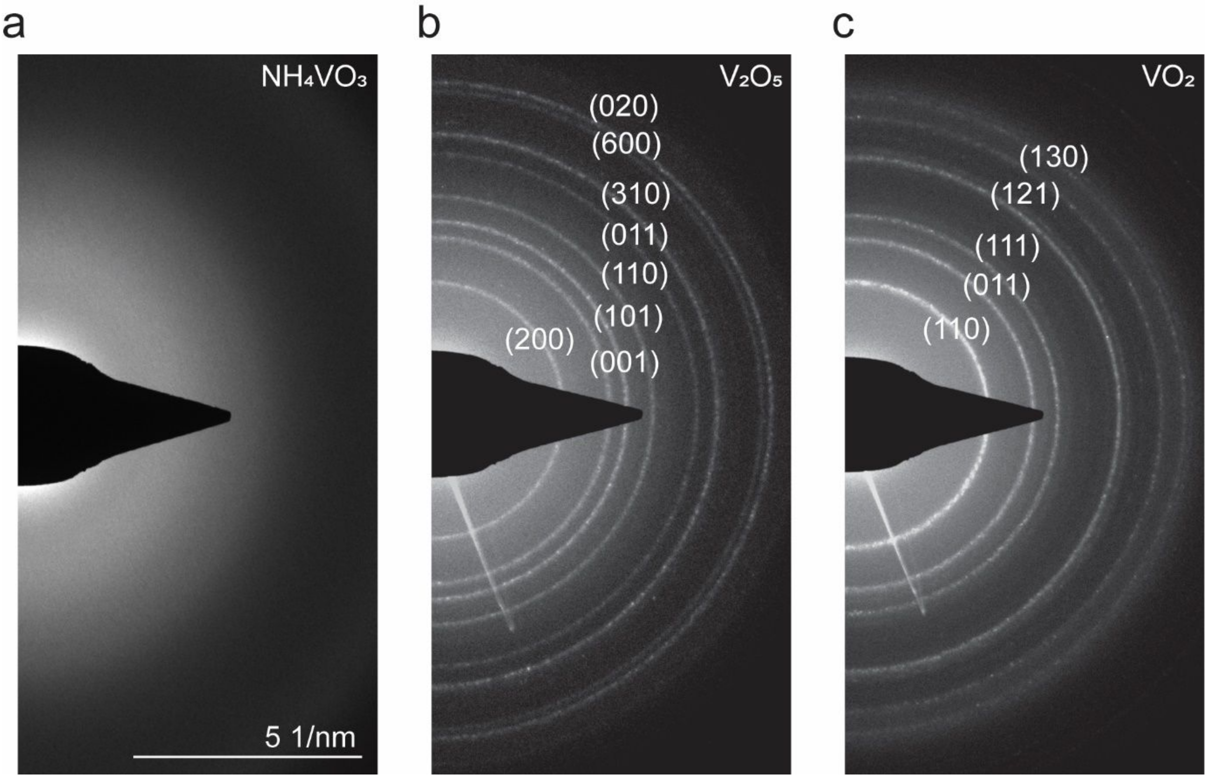

Figure S4. Electron diffraction patterns (DP) during the *In situ* TEM heating experiment. (a) DP of the amorphous ATT precursor at room temperature. (b) DP of the crystalline orthorhombic V_2O_5 at 500 °C. (c) DP of the crystalline rutile phase VO_2 at 700 °C.
